# Esophageal Stricture Caused by* Actinomyces* in a Patient with No Apparent Predisposing Factors

**DOI:** 10.1155/2019/7182976

**Published:** 2019-01-02

**Authors:** Allison N. Zhang, Debra Guss, Smruti R. Mohanty

**Affiliations:** Division of Gastroenterology and Hepatobiliary Diseases, New York-Presbyterian Brooklyn Methodist Hospital, Brooklyn, NY, 11215, USA

## Abstract

*Actinomyces* species are Gram positive anaerobic or microaerophilic bacteria that are part of the human flora in the oropharyngeal, gastrointestinal, and genitourinary tract. In the presence of a mucosal injury, they can become pathogenic and infect the underlying tissue without respect for tissue planes, leading to abscesses, fistulas, and sinus tracts. Through contiguous and hematogenous spread, virtually any organ can become infected. The presentation can be myriad and often mimics tumors, tuberculosis, or other more common infections and inflammatory conditions. While the cervicofacial region is the most common site of infection, involvement of the esophagus is unusual. Esophageal actinomycosis mostly occurs in patients with compromised immunity or prior esophageal injuries. Occurrence in immunocompetent individuals without risk factors is exceedingly rare. We report a case of esophageal actinomycosis complicated by an esophageal stricture in a patient with no apparent predisposing conditions.

## 1. Introduction

*Actinomyces* species are Gram positive anaerobic or microaerophilic filamentous bacteria belonging to the family Actinomycetaceae [1, 2]. These organisms are found in soil but also exist in humans as part of the oropharyngeal, gastrointestinal (GI), and genitourinary (GU) flora. They typically exhibit low virulence and slow growth, leading to chronic indolent infections that take months to manifest [1–3]. The most common site of infection in humans is the cervicofacial area, followed by the thorax, abdomen, and pelvis [1]. The most frequently isolated pathogenic species from these sites is* Actinomyces israelii*. Actinomycosis involving the esophagus is extremely rare [4]. Since the first reporting in 1929, fewer than 30 cases have been indexed in the English literature [4, 5]. The majority of esophageal actinomycosis occurs in patients with immunocompromising comorbidities or prior esophageal injuries [6]. Very rarely, it has also been observed in immunocompetent patients [6–9]. We report a case of esophageal actinomycosis complicated by an esophageal stricture in a patient with no clear underlying risk factors. To the best of our knowledge, this is the first case of its kind to be reported in the United States.

## 2. Case Report

**Figure 1:**
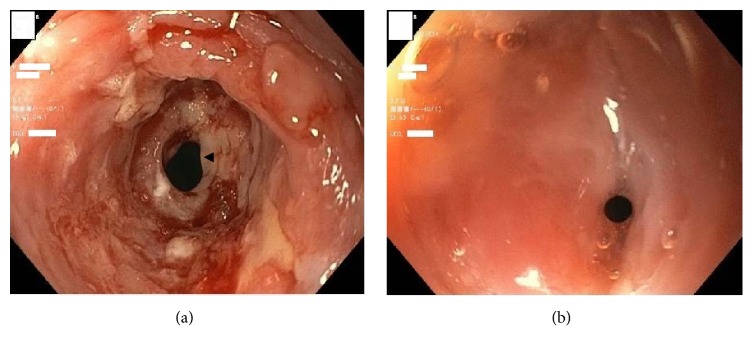
(a) Esophagogastroduodenoscopy (EGD) showing proximal esophageal inflammation and ulceration with a stricture at 15 cm from the incisors (arrowhead). (b) Repeat EGD showing healing of the esophageal mucosa and a remaining esophageal stricture.

**Figure 2:**
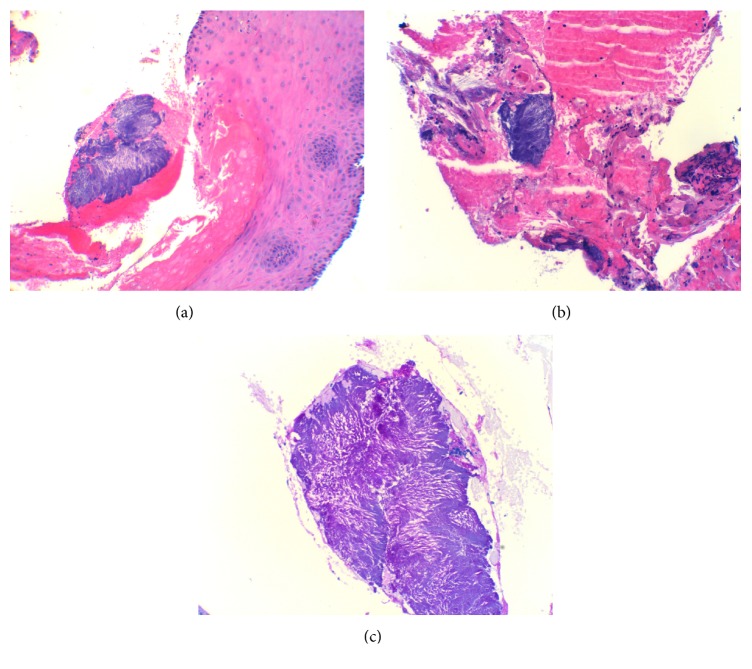
(a) Hematoxylin and eosin (H&E) stain showing esophageal squamous epithelium with hyperparakeratosis and hemorrhage secondary to* Actinomyces* presenting as sulfur granules (center blue). (b) H&E stain showing sulfur granules associated with esophageal epithelium degeneration, hemorrhage, neutrophils, and lymphocytes. (c) Periodic acid–Schiff stain showing organisms of* Actinomyces* presenting as sulfur granules and filaments.

A 45-year-old Haitian American female with no significant medical history presented with a six-month history of progressive solid food dysphagia and a one-month history of odynophagia. The patient had lost 15 pounds and was only able to tolerate pureed food or liquids. She had experienced no symptomatic relief on omeprazole 40 mg twice daily for the past two months and did not use tobacco, alcohol, or illicit substances. Her initial blood count and metabolic panel were unremarkable. An esophagogastroduodenoscopy (EGD) revealed erythematous and friable mucosa with ulcerations in the proximal esophagus (Figure 1(a)). There was a stricture encountered at 15 cm from the incisors through which the gastroscope could not be traversed. Biopsies were taken from the inflamed esophageal mucosa and the proximal lumen of the stricture. Brush cytology was collected through the stricture as there was a concern for malignancy. A subsequent barium esophagram and upper GI series demonstrated 2 cm irregular narrowing in the cervical esophagus, but no abnormalities in the rest of the esophagus, the gastroesophageal junction, stomach, duodenum, or proximal jejunum. Contrast-enhanced computed tomography (CT) of the chest showed no acute esophageal, mediastinal, pulmonary, or cardiac pathology. The esophageal biopsy indicated acute and chronic inflammation with filamentous sulfur granules consistent with* Actinomyces*; rare fungal hyphal elements were additionally identified (Figures 2(a), 2(b), and 2(c)). The cytology was negative for malignant cells and the acid-fast bacilli (AFB) stain was negative, ruling out* Nocardia* as a potential pathogen. The patient was started on intravenous (IV) Penicillin for a diagnosis of esophageal actinomycosis and oral Fluconazole for a presumed* Candida* coinfection given concurrent fungal elements. Her human immunodeficiency virus (HIV) status was negative and her fasting blood glucose was within the normal range. She was discharged on Fluconazole 200 mg daily for 2 weeks and IV Penicillin G 3 million units every 4 hours for 6 weeks followed by oral Penicillin to complete a total of 6 months of antibiotics. Her esophageal culture eventually grew normal oropharyngeal flora and rare* Candida albicans*. The patient returned to the hospital 6 weeks later due to an acute right upper extremity deep venous thrombosis (DVT) associated with her peripherally inserted central catheter (PICC). Her odynophagia had improved but she was still not able to advance her diet. Repeat EGD was performed, which showed resolution of her esophagitis, but a remaining stricture in the proximal esophagus (Figure 1(b)). During an attempt to dilate the stricture, a small mucosal tear was induced. The dilation had to be deferred at that point pending mucosal healing. A repeat barium esophagram illustrated the previously identified stricture with no perforation. The patient's PICC was removed and she was discharged on oral amoxicillin 875 mg twice daily to complete the 6-month course. The patient was given a follow-up appointment in our GI clinic where she would be reassessed for esophageal stricture dilation. Unfortunately, she was lost to follow-up.

## 3. Discussion

*Actinomyces* are human commensal bacteria that only trigger an infection in the presence of an impaired mucosal defense, often due to surgery, trauma, foreign instrumentation, inflammation, or immunocompromising comorbidities [4, 6, 10, 11]. It is rare to find* Actinomyces* as the sole culprit of an infection as most infections are revealed to be polymicrobial, involving additional pathogens such as* Actinobacillus*,* Eikenella*, Enterobacteriaceae,* Fusobacterium*,* Streptococcus*, or* Candida* [9, 10, 12, 13]. It is thought that these companion organisms may facilitate the introduction and propagation of* Actinomyces* at a target site [1, 11]. The mechanism of actinomycosis involves an antecedent disruption in the mucosal barrier, followed by infiltration of the bacteria into the submucosal layers and further invasion by the bacteria across the tissue planes, leading to the formation of sinus tracts, fistulas, or abscesses and contiguous seeding of the infection in neighboring organs [1, 10–12]. Translocation to distal targets can also occur via hematogenous dissemination [1]. Actinomycosis commonly engenders a chronic granulomatous inflammation associated with an intense desmoplastic response [1, 2, 11]. Due to an indolent progression, it may take months to years for symptoms of actinomycosis to manifest, and due to its varied presentation, it is frequently mistaken for other diagnoses such as malignancy, tuberculosis, or other more common infectious and inflammatory disorders [1, 3]. Virtually all organs can be affected, including the face, neck, thorax, abdomen, pelvis, central nervous system, joints, bone, and soft tissue [1].

Abdominal actinomycosis accounts for 25% of all actinomycosis, with ileocecal involvement being the most common followed by that of the left colon [1, 2, 11]. Preceding triggers include perforated appendicitis, diverticulitis, and intra-abdominal procedures [11]. Infections at other GI sites including the esophagus, liver, pancreas, biliary tract, and small intestine have all been reported [11]. Esophageal actinomycosis in particular has an exceptional occurrence [2, 4]. It can manifest as esophagitis, esophageal ulcerations, abscesses, fistulas, sinus tracts, or strictures [2, 4]. As with infections at other sites, there is usually a preceding mucosal injury and the culture is often polymicrobial. Although greater than 75% of the cases occur in immunocompromised patients, studies over a long term have not revealed an association between the incidence of esophageal actinomycosis and underlying compromised immunity [2, 6]. Our patient had no apparent predisposing factors for actinomycosis, although a* Candida* coinfection might have facilitated it. The esophageal stricture formation was likely a result of the desmoplastic response typical in actinomycosis.

On histology,* Actinomyces* appear as sulfur granules with club-shaped ends, which signify clusters of bacteria in a proteinaceous matrix [1, 10]. As* Nocardia* also exhibits such an appearance, an AFB stain is usually employed to differentiate between the two [14]. Culture remains the gold standard of diagnosis [1, 10]. Unfortunately, due to nonexpeditious transport of the specimen to a strict anaerobic medium, competing growth from coinfectious organisms, and prior antibiotic exposure, the yield of culture is very low, 24% in some studies [1, 2, 9]. This frequently obligates a diagnosis based on histology alone, in the appropriate clinical setting [10]. Furthermore, sulfur granules, although an essential component of actinomycosis, are only observed in 50% of cases, so their absence does not necessarily rule out actinomycosis [1, 11]. The treatment of actinomycosis typically consists of high dose IV Penicillin for 2-6 weeks followed by oral Penicillin for another 6-12 months, although shorter duration has been reported [1, 2, 15].* Actinomyces* remain highly sensitive to Penicillin; however, if Penicillin allergy is present, tetracycline and macrolides are alternative options [1, 2]. In the case of cutaneous actinomycosis, oral amoxicillin and clavulanic acid have been used in place of IV Penicillin with success [16]. Surgery is reserved for infections with a necrotic nidus, large infectious burden, fistulas, and abscesses [1, 6, 7]. Given that actinomycosis can mimic other diseases and a diagnosis can be delayed, clinicians should be vigilant about this rare but serious infection, especially in patients with impaired immunity, brakes in the mucosal lining of the GI or GU tract, and refractory symptoms despite initial therapy and even among immunocompetent individuals where the location of a disease may be atypical such as a peptic stricture occurring in the cervical esophagus in our case. If there is a clinical suspicion for actinomycosis, the appropriate means of diagnosis is to obtain a tissue biopsy and culture, while ensuring the timely transport of the specimen to an optimal culture environment.
